# Introduction of Water‐Vapor Broadening Parameters and Their Temperature‐Dependent Exponents Into the HITRAN Database: Part I—CO_2_, N_2_O, CO, CH_4_, O_2_, NH_3_, and H_2_S

**DOI:** 10.1029/2019JD030929

**Published:** 2019-11-07

**Authors:** Y. Tan, R. V. Kochanov, L. S. Rothman, I. E. Gordon

**Affiliations:** ^1^ Atomic and Molecular Physics Division Harvard‐Smithsonian Center for Astrophysics Cambridge MA USA; ^2^ Now at Hefei National Laboratory for Physical Sciences at Microscale, iChem Center University of Science and Technology of China Hefei China; ^3^ Laboratory of Quantum Mechanics of Molecules and Radiative Processes Tomsk State University Tomsk Russia

**Keywords:** HITRAN, line broadening, water vapor, radiative transfer, spectroscopy

## Abstract

The amount of water vapor in the terrestrial atmosphere is highly variable both spatially and temporally. In the tropics it sometimes constitutes 4–5% of the atmosphere. At the same time collisional broadening of spectral lines by water vapor is much larger than that by nitrogen and oxygen. Therefore, in order to accurately characterize and model spectra of the atmospheres with significant amounts of water vapor, the line‐shape parameters for spectral lines broadened by water vapor are required. In this work, the pressure‐broadening parameters (and their temperature‐dependent exponents) due to the pressure of water vapor for spectral lines of CO_2_, N_2_O, CO, CH_4_, O_2_, NH_3_, and H_2_S from both experimental and theoretical studies were collected and carefully reviewed. A set of semiempirical models based on these collected data was proposed and then used to estimate water broadening and its temperature dependence for all transitions of selected molecules in the HITRAN2016 database.

## Key Points

- Water‐vapor broadening parameters for molecules in the HITRAN database are introduced for the first time
- H_2_O‐broadening effects for spectral lines of different molecules are discussed
- Procedures describing how to work with new parameters using HITRAN*online* and HAPI are explained

## 1 Introduction

The current edition of HITRAN2016 (Gordon et al., 2017) has substantially increased the potential for the database to model radiative processes in terrestrial and planetary atmospheres. The previous editions of the database (from HITRAN2004 (Rothman et al., 2005) to HITRAN2012 (Rothman et al., 2013)) provided a limited set of parameters for each line transition, fixed within a 160‐character record in ASCII files. Within that parameterization, only self‐ and air‐broadening parameters (which in HITRAN formalism are half‐widths at half maximum (HWHM) provided at 296 K and 1 atm of pressure), and the temperature dependence of the air‐broadening HWHMs for the line list of each HITRAN molecule were available. The new edition has introduced line broadening parameters due to the pressure of H_2_, He, and CO_2_ for molecules of planetary interest including SO_2_, NH_3_, HF, HCl, OCS, and C_2_H_2_ for the first time (Wilzewski et al., 2016). Subsequently, the corresponding data for the CO molecule broadened by planetary gases was added from Li et al. (2015). This has instigated a significant progress for generating high‐precision molecular absorption cross sections relevant to the studies of planetary atmospheres. This new initiative took full advantage of the new structure of the HITRAN database (see Hill et al., 2016) which allows storage and effective retrieval of these parameters. The HITRAN Application Programming Interface (HAPI; Kochanov et al., 2016) also makes good use of these new parameters allowing the calculation of cross sections at different proportions of ambient gases (see Figure 31 of the HITRAN2016 paper (Gordon et al., 2017) for instance).

In this work we continue to build on this success and add broadening parameters due to the pressure of water vapor for the lines of CO_2_, N_2_O, CO, CH_4_, O_2_, NH_3_, and H_2_S.

**Figure 1:**
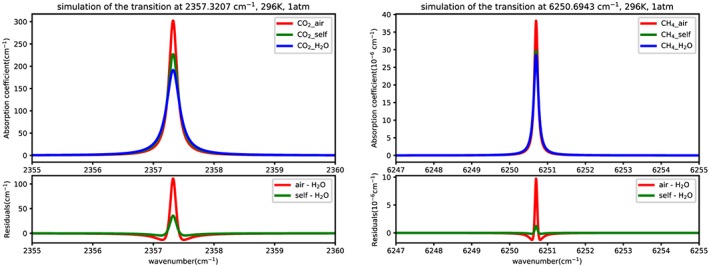
Comparison of simulated absorption cross sections for the CO_2_ transition at 2,357.3207 cm^−1^ and the CH_4_ transition at 6,250.6943 cm^−1^ broadened by air, self, and H_2_O. The bottom panel represents the difference in absorption coefficients calculated with air‐ and self‐broadening to that with water‐vapor broadening.

It is well known that water vapor is highly variable in Earth's atmosphere, and it is also been confirmed in recent studies (Benneke & Seager, 2013; Hedges & Madhusudhan, 2016) that water vapor can represent a potentially significant cross‐sensitivity source in exoplanets. Water vapor is a major absorber of the infrared light in the terrestrial atmosphere but it is also a very efficient broadener of spectral lines for other gases. The broadening by water vapor is much larger than that of nitrogen and oxygen which are the two main contributors to dry air broadening. Figure 1 shows the simulated cross sections for the CO_2_ R(10) transition in the ν_3_ band at 2,357.3207 cm^−1^ and CH_4_ transition at 6,250.6943 cm^-1^ broadened by air‐, self‐, and H_2_O using HAPI (Kochanov et al., 2016). Therefore, although nitrogen and oxygen are the most abundant terrestrial gases, water vapor does make an appreciable impact on the retrievals, especially in the tropics where water concentrations reach up to 5%. For instance, satellite‐based space projects including the NASA Orbiting Carbon Observatory re‐flight (OCO‐2) (Crisp, 2015), as well as the more recent OCO‐3 (Eldering et al., 2019), the TANSO‐FTS on the Japanese Green‐house Gases Observing satellite (GOSAT; Kuze et al., 2009) and the Chinese TanSat satellite instrument (Chen et al., 2012) are proposed to retrieve surface pressure and column abundances of CO_2_ with subpercent precision. Central to the accuracy of remote‐sensed CO_2_ quantities, however, is the accuracy of the spectroscopic input (line positions, line intensities, and line‐shape parameters) used in atmospheric models within the retrieval algorithms. The parameterization of the line shape includes pressure broadening and shift parameters, as well as their temperature‐dependent exponents. Besides, they may also include some additional physical phenomena affecting line shape such as line mixing, line narrowing, and speed dependence. Since the ambient atmospheric surface pressure of water vapor cannot be neglected due to its strong broadening efficiency, reducing uncertainties associated with water vapor is imperative to achieve a subpercent precision in high‐precision remote sensing for CO_2_ retrieval (C. E. Miller et al., 2005). Interestingly, the atmospheres of rocky planets that may have suffered large impacts are expected to have “steamy” atmospheres (Benneke & Seager, 2013), and therefore, knowledge of broadening of spectral lines by water vapor is important for modeling spectra of these atmospheres. In their recent paper, (Gharib‐Nezhad & Line, 2019), emphasized the importance of using proper water broadening parameters when modeling the emission and transmission spectra as well as on the vertical energy balance in sub‐Neptune/super‐Earth atmospheres.

Water‐vapor broadening parameters (γ_H2O_) as well as their temperature‐dependent exponents (n_H2O_) have been collected from both experimental and theoretical studies. The collected data were used to create semiempirical models so we could populate the entire line lists of relevant molecules in the HITRAN2016 database with relevant parameters. Considering that the uses of these parameters may go beyond traditional HITRAN applications and could be used to model combustion processes or “hot” planetary atmospheres, we want these models to be applicable for the forthcoming updates (partially explained in Hargreaves et al. (2019) and Li et al. (2015)) of the high‐temperature database analog to HITRAN, HITEMP (Rothman et al., 2010) as well. Therefore, wherever there was enough experimental and theoretical data available, the models were based on the Padé approximants which allow smooth extrapolation to the high‐rotational levels rather than using polynomials or fixing the widths of the high‐*J* lines to the value of the last measured transition which was the case in the previous efforts (for instance in Wilzewski et al. (2016) and Li et al. (2015)). Associated programs in Python that allow calculating the water‐vapor broadening parameters for CO_2_, CO, and O_2_ even at very high *J*'s are provided in the supporting information. The semiempirical models derived here can be applied to all isotopologues of the molecules in question.

## 2 Generated Data Sets

The line‐shape parameters derived in this paper still follow the traditional HITRAN “.par” formalism where Lorentzian half‐widths (at T_ref_ = 296 K) are provided based on the Voigt profile measurements. The sophisticated line shapes beyond the Voigt profile (Wcisło et al., 2016) were not considered in this work since the majority of the water broadening parameters that came from measurements and calculations in the literature was still based on the Voigt profile. The γ_H2O_ and n_H2O_ parameters can be retrieved from HITRAN*online* (http://www.hitran.org) in user‐defined formats (see section 4 for details). The conversion of the half‐width from a reference temperature (T_ref_) can be described by the following power law:

(1) $$\gamma \left(T\right)=\gamma \left(T_{\mathrm{ref}}\right)\cdot {\left(\frac{T_{\mathrm{ref}}}{T}\right)}^{n}$$

Here *n* is the temperature‐dependent exponent which could also be determined from the slopes of the least‐square fits of −ln*γ*(*T*) vs. ln*T*. It then leads to

(2) $$n_{H_{2}O}=-\frac{\ln\gamma_{H_{2}O}\left(T_{\mathrm{ref}}\right)-\ln\gamma_{H_{2}O}\left(T\right)}{\ln T_{\mathrm{ref}}-\ln T}$$

It is also worth emphasizing that the temperature‐dependent exponents, derived from the power law above, work only within a relatively narrow temperature regime. A recent study also shows that a double power law developed by (Gamache & Vispoel, 2018) can model the temperature dependence of the half‐widths over large temperature ranges. However, the data available for this fitting procedure are quite limited, and consequently we have chosen one temperature exponent at 296 K in this work.

### 2.1 CO_2_

After water vapor, carbon dioxide is the second strongest absorber of infrared radiation (including thermal IR) despite its low concentration in the Earth's atmosphere. This makes it an important greenhouse gas which is monitored by many ground‐based and satellite‐based instruments as mentioned above. In addition, there are many other satellite missions, for instance, the Atmospheric Infrared Sounder (AIRS), the Infrared Atmospheric Sounding Interferometer (IASI), the Tropospheric Emission Spectrometer (TES), and the Cross‐track Infrared Sounder (CrIS) missions all reporting CO_2_ measurements in the thermal infrared. The OCO‐2 mission was proposed to reach the subpercent precision of CO_2_ concentration measurement which places extraordinary demand on the quality of spectroscopic parameters of CO_2_ lines. The pressure broadening by water vapor in the tropical regions makes an appreciable contribution to the measured widths of CO_2_ lines and therefore needs to be accounted for when such stringent requirements are placed on the atmospheric retrievals. It is thus important to introduce accurate half‐widths as well as temperature‐dependent exponents of the half‐widths to the HITRAN database in order to model CO_2_ absorption cross sections for high‐accuracy remote‐sensing tasks to avoid the spectroscopic uncertainties associated with ambient water vapor.

A theoretical calculation for CO_2_ broadening by water vapor has been made by Rosenmann, Hartmann, et al. (1988). The semiclassical Robert‐Bonamy formalism was used to predict γ_H2O_ for CO_2_ lines as well as their temperature‐dependent exponents at a wide temperature range from 300 to 2,400 K. It was demonstrated that for CO_2_, γ_H2O_ can reach twice the values of the air‐broadening half‐widths. Prior to the 21^st^ century, direct measurements were very rare and existed for only two lines (R(42) and R(54) in the ν_3_ band; Rosenmann, Perrin, et al. (1988). With increased requirements on the accuracy of atmospheric retrievals, more experimental data for CO_2_ broadening by water vapor have recently emerged. The γ_H2O_ parameters for 182 ^12^CO_2_ lines in the ν_3_ and ν_2_+ν_3_‐ν_2_ bands, as well as the ν_3_ band of ^13^CO_2_, were reported by Sung et al. (2009) with a standard Voigt profile near room temperature. There were three transitions of CO_2_ broadened by water vapor near 1.57 μm that had been measured using speed‐dependent Voigt profile near room temperature (Wallace et al., 2011) which were not included in our fitting procedure because of the poor agreement with other measurements and relative large uncertainties. More recently, Delahaye et al. (2016a) have reported the transmission spectra of CO_2_ in a high concentration of water vapor, also in the 4.3‐μm region. The γ_H2O_ parameters for 64 CO_2_ transitions were determined at 323 and 367 K (Delahaye et al., 2016a). All these theoretical and experimental results were used to generate the data set for CO_2_ transitions appropriate for the HITRAN database.

**Figure 2:**
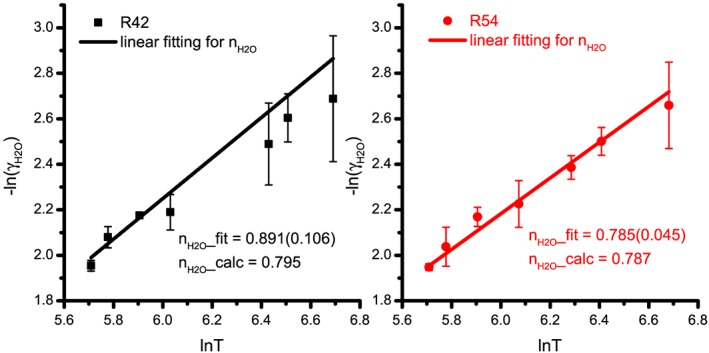
The temperature‐dependent exponents, *n*_H2O_, for the R(42) and R(54) transitions in the ν_3_ band of CO_2_ determined from the slopes of the least squares fits of −ln*γ*(*T*) vs ln*T* from 300 to 805 K (Delahaye et al., 2016a; Rosenmann, Hartmann et al., 1988; Rosenmann, Perrin, et al., 1988; Sung et al., 2009). The temperature‐dependent exponents derived from linear fitting agree well with the calculated parameters from Rosenmann, Hartmann et al. (1988).

**Figure 3:**
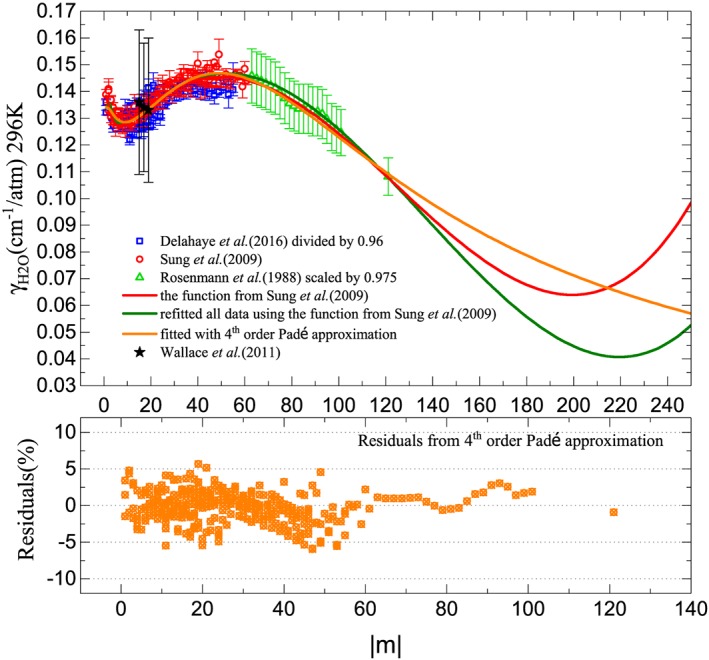
The top panel shows γ_Η2Ο_ parameters for CO_2_ transitions at 296 K based on the empirical fitting results from both experiments and calculations. The fitted half‐widths from different functions are shown here in solid lines of different colors which are valid up to∣*m* ∣  ≤ 121. The bottom panel shows the relative residuals fitted from the third‐ to fourth‐order Padé approximation with a standard deviation of 3%.

Based on the comparison of the temperature‐dependent exponents from the linear fitting of the power law and that of the semiclassical calculations, the theoretical predictions for the temperature dependences were used as shown in Figure 2. The experimental results on γ_H2O_ for CO_2_ from Delahaye et al. (2016a) at 323 and 367 K, (Sung et al., 2009) near room temperature 301 K, and Wallace et al. (2011) at 294 K were carefully converted to corresponding values at 296 K by using temperature‐dependent exponents from the predictions of Rosenmann, Hartmann et al. (1988). Due to the large error bar for experimental results by Wallace et al. as well as the difference of the line profile used, these data were excluded from the final fitting procedure. There was about a 4% unexplained systematic offset between the measurements of Delahaye et al. (2016a) and those of Sung et al. (2009) in the same spectral region of 4.3 μm, although they were sharing the same rotational dependence. Sung et al. (2009) data are used for spectroscopic input for the OCO‐2 mission (Oyafuso et al., 2017), and therefore for consistency, the experimental results converted from (Delahaye et al., 2016a) were then divided by 0.96 before fitting. In order to make more accurate predictions for transitions involving high‐rotational quanta, the calculated half‐widths from Rosenmann, Hartmann et al. (1988) were also incorporated into the fitting (only for 63 ≤  ∣ *m* ∣  ≤ 101 and *m* = 121) but were multiplied by a factor of 0.975 derived here to account for discrepancy between these calculations and measurements from Sung et al. (2009). Here *m* is a running number which (when expressed as a function of lower state rotational quantum number *J″*) is equal to *−J″* for P‐branch transitions and *J″* + 1 for the R‐branch. Sung et al. (2009) fitted their data to equation (3) and the set of coefficients recommended in their work is listed in Table 1. We also use the same function to refit all the data collected from experiments and calculations as shown in Figure 3 and Table 1.

(3) $$\gamma_{H2O}\left(\left|m\right|\right)=\frac{a}{m^{2}}+\frac{b}{\left|m\right|}+c+d\cdot \left|m\right|+e\cdot m^{2}+f\cdot {\left|m\right|}^{3}$$

**Table 1:** Fitted Coefficients (From Equations (3) and (4)) for Calculating Water‐Vapor‐Broadened Lorentz Half‐Widths of CO_2_ (at 296 K and in the Units of cm/atm)

Coefficients (equation (3))	Sung et al. (2009)	This work	Coefficients (equation ((4)))	This work
*a*	−0.03563	−0.06539	*a*_0_	−25.18047
*b*	0.06318	0.09205	*a*_1_	459.98114
*c*	0.1093	0.10547	*a*_2_	35.11405
*d*	0.001498	0.00161	*a*_3_	0.67148
*e*	−1.848E − 5	−1.87E − 05	*b*_1_	3141.5695
*f*	4.924E − 8	4.58E − 08	*b*_2_	369.17545
			*b*_3_	−0.75135
			*b*_4_	0.05441
Valid range	|*m*| ≤ 121	|*m*| ≤ 121		|*m*| ≤ 121

In addition, the third‐ to fourth‐order Padé approximant was also used to fit all data points as described here.

(4) $$\gamma_{H2O}\left(\left|m\right|\right)=\frac{\left(a_{0}+a_{1}\cdot \left|m\right|+a_{2}\cdot m^{2}+a_{3}\cdot {\left|m\right|}^{3}\right)}{\left(1+b_{1}\cdot \left|m\right|+b_{2}\cdot m^{2}+b_{3}\cdot {\left|m\right|}^{3}+b_{4}\cdot m^{4}\right)}$$

The advantage of using this new function is that it extends standard Taylor treatments, overcoming convergence issues at *m* values beyond those measured in experiments which could be very important at high temperatures. As shown in Figure 3, when it comes to high *m* values, the predictions from the polynomial function is increasing unphysically after |*m*| = 180 while the Padé approximant provides more reliable values. The fitting residuals scatter within 5% between our fitted values and the experimental results. Although the difference of these two functions (both polynomial function from Sung et al. and Padé approximant) for *m* lower than 121 is negligible, the Padé approximant is more reliable for”hotter” environments such as exoplanetary atmospheres or combustion processes where one can observe transitions with high *m* values.

In conclusion, the vibrational dependence of the γ_H2O_ for CO_2_ is almost negligible based on the available data, which is typical for linear molecules. Therefore, when estimating γ_H2O_ of CO_2_ one should be concerned only with the rotational distribution which could be described from the third‐ to fourth‐order Padé approximant as shown by equation (4), with coefficients from Table 1. The temperature‐dependent exponents for |*m*| less than 101 are taken from theoretical calculations by Rosenmann, Hartmann et al. (1988) and complemented with extrapolated values. The constant value 0.63 was used for |*m*| greater than 101.

### 2.2 N_2_O

Nitrous oxide is one of the important air pollutants. Therefore, the concentration of N_2_O in the Earth's atmosphere has also been monitored by both ground‐based and satellite‐based instruments in recent years (Kangah et al., 2018; Wunch et al., 2010; Xiong et al., 2014; Zhou et al., 2019). The water‐vapor broadening parameters of N_2_O lines are needed for high‐accuracy retrieval to determine their concentrations. To the best of our knowledge, this has so far not been widely considered by atmospheric remote sensing. Unfortunately, only one water‐broadened transition of N_2_O has been measured (Deng et al., 2017). The case of N_2_O should be very close to that of CO_2_ since their molecular electrostatic surface potential is very similar, as well as the signs and magnitudes of their electric quadrupole moment. The calculated and experimental intermolecular distances reported by Alkorta and Legon (2018) for N_2_O … H_2_O and CO_2_ … H_2_O are very close. Therefore, γ_H2O_ for CO_2_ were used to represent those for N_2_O using a scaling factor of 0.92 derived using the corresponding experimental value from (Deng et al., 2017).

### 2.3 CO

The water‐vapor broadening parameters of carbon monoxide lines are needed for the high‐accuracy trace gas retrieval missions (e.g., Clerbaux et al., 2008; Liu et al., 2011, 2014), and these parameters are also very useful in the study of exoplanets with substantial amounts of water vapor in the atmosphere (Konopacky et al., 2013). The water‐vapor broadening coefficients of CO transitions in the fundamental band have been measured by Deng et al. (2017), Soufiani and Hartmann (1987), Willis et al. (1984), and Varghese and Hanson (1981). (Henningsen et al. (1999) performed water‐vapor broadening measurement for the R(7) transition in the 3‐0 overtone band. The complete calculation of γ_H2O_ for CO lines in the 200–3,000‐K temperature range had been presented by Hartmann et al. (1988). They were made with a semiclassical model derived from the Robert and Bonamy (RB) approach.

**Figure 4:**
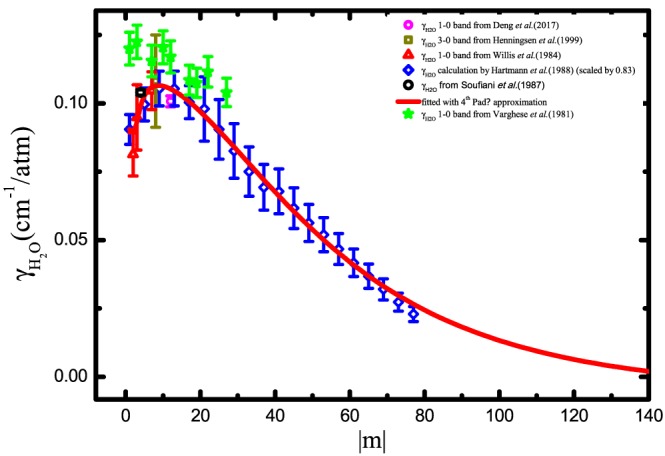
γ_H2O_ for CO lines. The magenta circle represents the broadening parameters for the R(11) transition in the fundamental band measured by (Deng et al., 2017); red triangles—the P(2), P(3), and P(7) transitions in the fundamental band are from (Willis et al., 1984); yellow square—the R(7) transition in the second overtone band by (Henningsen et al., 1999); green stars—measurements in the fundamental band from (Varghese & Hanson, 1981); and black circle—P(4) transition from (Soufiani & Hartmann, 1987). The blue triangles were scaled calculations from (Hartmann et al., 1988) and the red line corresponds to the fitting result of the third‐ to fourth‐order Padé approximant.

The calculations were found to be systematically higher than those obtained from experiments (except for data from Varghese and Hanson (1981) which were found to be in poor agreement with other experiments) by about 20%. Therefore, the calculated values were multiplied by the factor 0.83 according to our comparison with experimental results as shown in Figure 4. The third‐ to fourth‐order Padé approximant was applied to fit the data from both theoretical values and experimental results while constraints were made at very high rotational levels to avoid negative results during the fitting. Therefore all data reported in Deng et al. (2017), Hartmann et al. (1988), Henningsen et al. (1999), Soufiani and Hartmann (1987), and Willis et al. (1984) were fitted with a least squares procedure to a Padé approximant (see equation (4)). The fitting resulted in the following coefficients: *a*_0_ = 8.68258, *a*_1_ =  − 20.7531, *a*_2_ = 12.83443, *a*_3_ =  − 0.08283, *b*_1_ =  − 95.99764, *b*_2_ = 102.62357, *b*_3_ =  − 0.12397,*b*_4_ = 0.0238. Then the fitting results based on equation (4) were used with |*m*| ≤ 150, and the HITRAN uncertainty code is increasing with rotational quantum number from 5 to 3 (see definition of HITRAN uncertainty codes in the documentation pull‐down menu in HITRAN*online*; https//http://hitran.org/docs/uncertainties). For |*m*| > 150, a constant value 0.001 was used. The temperature‐dependent exponents from the calculation in Hartmann et al. (1988) were interpolated to cover the entire region with |*m*|≤ 77. For |*m*|> 77, the constant value of 0.26 corresponding to *n* at |*m*|= 77 was used to apply to the rest of the lines.

### 2.4 CH_4_

Following water vapor and carbon dioxide, methane is also one of the most significant greenhouse gases in the atmosphere, and its warming potential is almost 23 times that of carbon dioxide over a 100‐year cycle (Solomon et al., 2007). Therefore, monitoring methane in the terrestrial atmosphere is one of the main objects in many remote‐sensing missions including GOSAT (Kuze et al., 2009), SCIAMACHY (Frankenberg et al., 2011), MERLIN (Ehret et al., 2017), CarbonSat (Buchwitz et al., 2013), and TROPOMI (Butz et al., 2012). For the purpose of reducing the uncertainties in the retrieval of atmospheric methane column amounts, it requires one to model the absorption cross sections of methane with an extremely high accuracy (to even subpercent). It is therefore necessary to consider the contribution of the pressure broadening of CH_4_ by water vapor besides the self‐ and air‐broadening contributions (McDermitt et al., 2011; Miller et al., 2015). There were an appreciable amount of methane lineshape laboratory studies and theoretical calculations in recent years, but almost all of them were devoted to self‐ and/or air‐broadening line‐shape parameters of methane (see for instance, Devi et al., 2015, 2016; Ghysels et al., 2014; Hashemi et al., 2015; Smith et al., 2014). The γ_H2O_ parameters for CH_4_ transitions in the mid‐infrared and near‐infrared region were measured recently for the first time using a Fourier Transform spectrometer (Delahaye et al., 2016c). In that study 76 ro‐vibrational transitions were measured. Prior to that, only the 2ν_3_ R(3) and R(4) manifolds located in the 1.6‐μm region were studied from 316 to 580 K by diode laser absorption spectroscopy in (Gharavi & Buckley, 2005), and the ν_4_ P(5), P(9), and P(10) manifolds located in the 8‐μm region were measured by Tunable Diode Laser Absorption Spectrometer at room temperature in Lübken et al. (1991).

**Figure 5:**
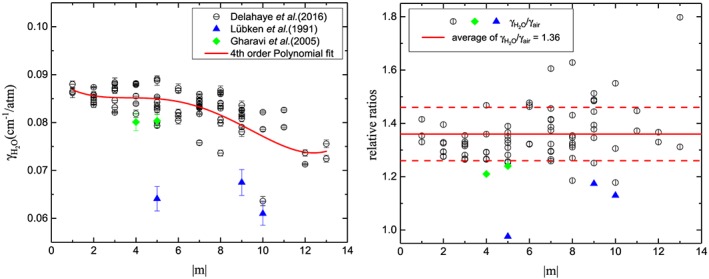
γ_H2O_ for methane. (a) Black circles represent the water‐vapor broadening Lorentz half‐widths of CH_4_ in the mid‐infrared and near‐infrared region by (Delahaye et al., 2016c) with the red line for the fourth‐order polynomial fitting (not used in this work), blue triangles from the ν_4_ P(5), P(9), and P(10) manifolds by Lübken et al. (1991), and the 2ν_3_ R(3) and R(4) manifolds in green diamonds by Gharavi and Buckley (2005). (b) The ratio of the water‐vapor broadening half‐widths of methane transitions to their corresponding air‐broadening half‐widths in HITRAN, as well as the average value of the those ratios plotted here.

In general, the measured γ_H2O_ of methane lines were found to be about 30% larger than their air‐broadening parameters as shown in Figure 5. Since the rotational dependence of the water‐vapor broadening effects (including the effect of rotational symmetry) for methane is still not quite clear based on our analysis of the available measurements, the water‐vapor broadening coefficients at room temperature were then generated from the scaled value of their corresponding air‐broadening parameters. The scaling factor of 1.36 (with a standard deviation of 0.10) was used based on the measurements from Delahaye et al. (2016c), Lübken et al. (1991), and Gharavi and Buckley (2005) to their corresponding air‐broadening parameters in HITRAN. As for their temperature‐dependent exponents, the scaling factor of 1.26 to the air‐broadening temperature‐dependent exponents was used as well, which also came from the experimental measurement of the 2ν_3_ R(3) and R(4) manifolds in Gharavi and Buckley (2005).

### 2.5 O_2_

Molecular oxygen is the second most abundant gaseous component in the terrestrial atmosphere, and it is well‐mixed in air. In many ground‐based, airborne, or satellite‐based remote sensing projects (Blackwell et al., 2001; Cadeddu et al., 2007; Chen et al., 2012; Crisp, 2015; Kuze et al., 2009; Rosenkranz, 2001), the O_2_ 60 GHz and A‐band at 0.76 μm are being used extensively to provide the information on atmospheric path length and surface pressure. It was also demonstrated recently that the 1.27‐μm band can also be successfully employed in remote‐sensing missions (Sun et al., 2018). Since the accuracy of the retrievals relies directly on the accuracy of the spectroscopic parameters from the input line‐shape information, the broadening parameters of the lines are of crucial importance to these remote‐sensing missions. Therefore, it would be necessary to consider the pressure broadening of O_2_ lines by water vapor in addition to the self‐ and air‐broadening in order to reduce uncertainties in the spectroscopic input.

**Figure 6:**
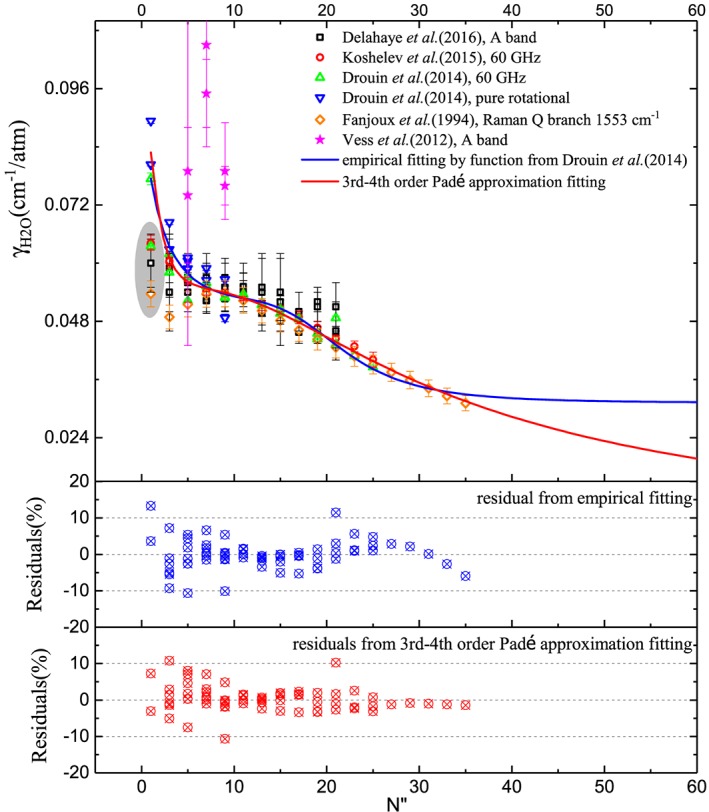
The top panel shows γ_H2O_ for O_2_ transitions at 296 K based on the fitting of the results from different experiments. The fitted half‐widths from different functions are shown here in solid lines of different colors which are valid up to |*m*|= 35. The bottom panel shows the residuals fitted from the empirical function of (Drouin et al., 2014) and the third‐ to fourth‐order Padé approximant with a standard deviation of 4% and 3%. Note here the grey‐shaded area in the top panel corresponds to the transitions with *N* ″  = 1 that were not included in our fitting procedure.

Unlike air‐ and self‐broadening in the O_2_ bands which have been studied extensively in recent years (Barnes & Hays, 2002; Drouin, 2007; Gordon et al., 2011; Long et al., 2010), only a few measurements have been performed for O_2_ perturbed by water vapor. In 1994, Fanjoux et al. (1994) presented the first extensive measurements of O_2_‐H_2_O broadening for the O_2_ Raman Q‐branch at 1,553.3 cm^−^ in a wide temperature range (between 446 and 990 K). Although the primary goal of that study was to support Raman thermometry of rocket engines, the authors had extrapolated their data to room temperature values based on the temperature‐dependent exponents fitted from the power law which would be comparable to other spectral regions. In recent years, a few more studies dedicated to water‐vapor broadening parameters of O_2_ lines in the pure rotational and A‐band were made by using different techniques: a laser‐based photoacoustic spectrometer (Vess et al., 2012), a frequency‐multiplier spectrometer with a Zeeman‐modulated absorption cell (Drouin et al., 2014), a radio‐acoustic detection spectrometer (Koshelev et al., 2015), and a Fourier transform spectrometer (Delahaye et al., 2016b). In order to provide accurate and reliable spectral parameters for atmospheric applications, the water‐vapor broadening parameters of O_2_ lines in the HITRAN database were then divided into two parts for generating the data set. As the first part, γ_H2O_ for O_2_ lines in the A‐band were obtained by multiplying air‐broadening coefficients in the A‐band from the HITRAN2016 database by a single scaling factor of 1.1, as per recommendation by the most recent study from Delahaye et al. (2016b). For the rest of the transitions, a complete analysis for all collected experimental data (presented in Figure 6) was carried out. Note that the transitions with *N* ″  = 1 were not included in our fitting procedure (as shown in the grey‐shaded area of Figure 6) except for the pure rotational transitions from Drouin et al. (2014). Their values fall off the pattern due to the large spin splitting in the lowest rotational level. A special case has been made to reproduce the γ_H2O_ of O_2_ with *N* ″  = 1 by the average value of all available experimental results accounting the lowest rotational level. The empirical function of Drouin et al. (2014) (see equation (5)) and the third‐ to fourth‐order Padé approximant were applied to fit all the other collected data with results listed in Table 2 for both functions.

(5) $$\gamma \left(N"\right)=A_{\gamma }+\frac{B_{\gamma }}{\left(1+c_{1}\cdot N"+c_{2}\cdot N{"}^{2}+c_{3}\cdot N{"}^{4}\right)}$$

**Table 2:** Fitted Coefficients (From Equations (4) and (5)) for Calculating Water‐Vapor Broadened Lorentz Half‐Widths of O_2_ (at 296 K and in the Units of cm/atm)

Coefficients (equation (5))	Drouin et al. (2014)	This work	Coefficients (equation ((4)))	This work
*A*_*γ*_	1.409	0.031	*a*_0_	−5.19194
*B*_*γ*_	1.854	0.063	*a*_1_	7.43635
*c*_1_	0.382	0.383	*a*_2_	3.35387
*c*_2_	−2.50E − 2	−2.30E − 2	*a*_3_	0.40734
*c*_3_	4.06E − 5	3.26E − 5	*b*_1_	−46.17
			*b*_2_	116.8574
			*b*_3_	0.43672
			*b*_4_	0.35434
Valid range	∣*m* ∣ ≤ 35	∣*m* ∣ ≤ 35		∣*m* ∣ ≤ 121

*Note*. The transitions with *N* ″  = 1 were not included in the fitting procedure and were treated separately in the final data set generation.

The n_H2O_ were fitted from data in Fanjoux et al. (1994) and from Appendix A of Drouin et al. (2014). For *N* ″  ≤ 35, the fitted and interpolated values used were based on the results of Table 4 from Drouin et al. (2014), and the constant value 0.60 was used for the transitions with *N* ″  > 35.

### 2.6 NH_3_

**Figure 7:**
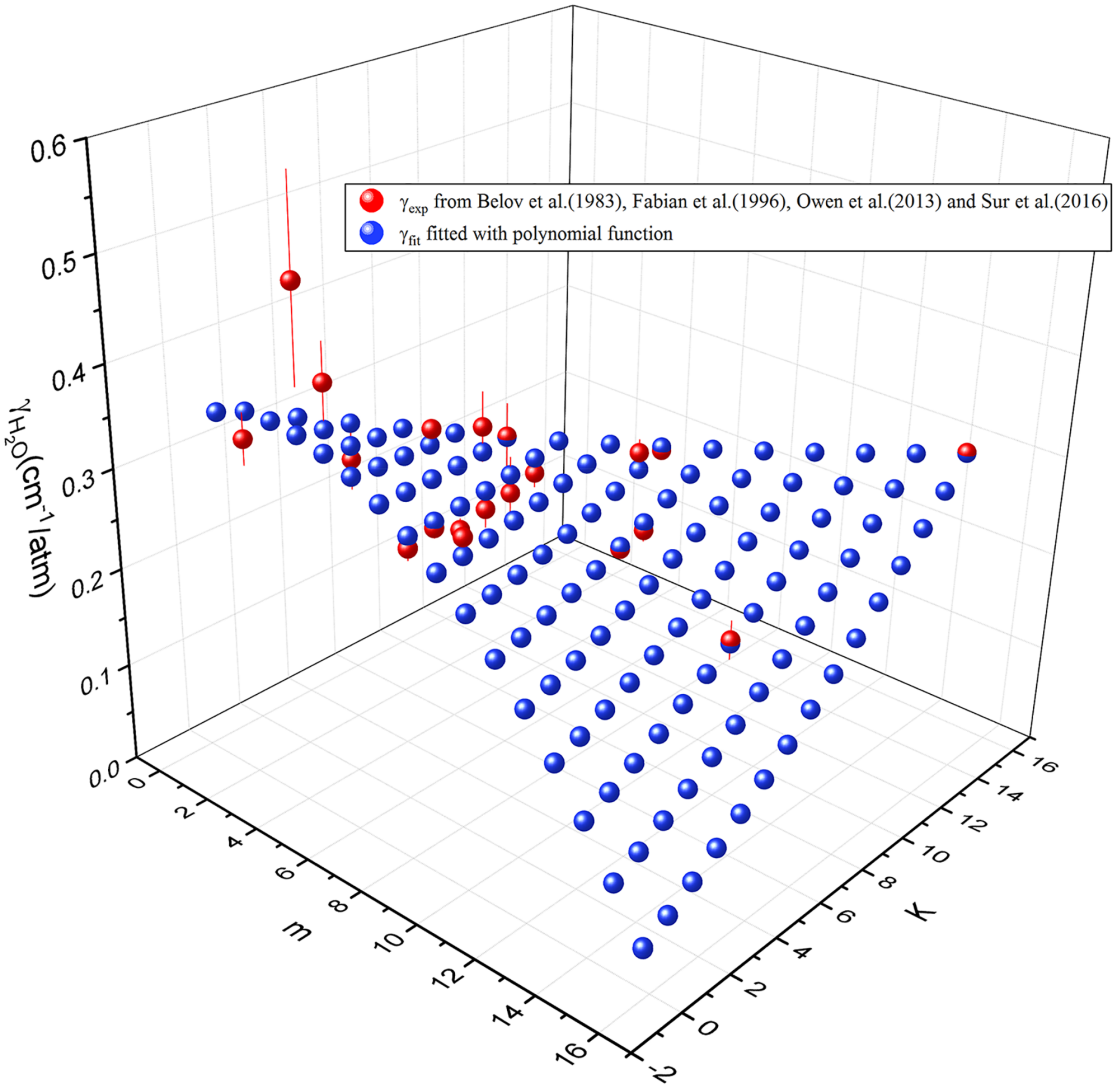
The pressure‐broadening coefficients of NH_3_ perturbed by water vapor versus the running index *m* and quantum numbers *K*. The red points correspond to experimental values taken from Fabian et al. (1996), Owen et al. (2013), and Sur et al. (2016), and the blue points correspond to the least squares fitting to the polynomial function (equation (6)).

Ammonia is a significant trace gas and is a subject of studies of different remote‐sensing missions (e.g., Beer et al., 2008; Höpfner et al., 2016). Experimental studies under Jovian conditions (Devaraj et al., 2014) suggested that broadening of ammonia lines by water can make an important difference when interpreting spectra from gas giants. Its accurate spectroscopic parameters are needed for a variety of gas‐sensing applications including those for environmental monitoring, industrial process control, and human breath analysis in medical science (e.g., Manne et al., 2006; Owen & Farooq, 2014; Schilt et al., 2004). While each of these applications involved significant concentrations of water vapor in the system, the impact of water vapor on ammonia absorption features is not negligible for high‐sensitivity detections of ammonia to ppb or ppm levels (D. J. Miller et al., 2015; Sun et al., 2015, 2017). In fact, recent experimental results from Schilt (2010) had demonstrated that water vapor was a significant cross‐sensitivity source especially in a high‐temperature environment. However, not quite enough data concerning water‐vapor broadening coefficients of NH_3_ lines were available in the literature. There is only one pure rotational line for which γ_H2O_ was measured (Belov et al., 1983). The water‐vapor broadening parameters of the NH_3_ strong *v*_2_ vibrational band around 10 μm have been measured by Fabian et al. (1996) and Owen et al. (2013). More recently, Sur et al. (2016) also published the water‐vapor broadening coefficients of NH_3_ Q‐branch transitions in the *v*_2_ vibrational band. Therefore, there were 19 transitions that had been studied in total as shown in Figure 7.

The dependence on the rotational quantum numbers for both *m* and *K* was considered in the fitting procedure based on the following equation:

(6) $$\gamma \left(m,K\right)=a_{0}+a_{1}\cdot m+a_{2}\cdot K+a_{3}\cdot m^{2}+a_{4}\cdot K^{2}+a_{5}\cdot m\cdot K$$

γ_H2O_ for ammonia versus the quantum numbers *m* and *K* as shown in Figure 7 help us to visualize the rotational dependence of the broadening coefficients, as they generally decrease with *m* but increase with *K*. The data reported in Belov et al. (1983), Owen et al. (2013), and Sur et al. (2016) were then fitted with a least squares procedure to a polynomial function, namely, equation (6). The values for these coefficients are *a*_0_ = 0.35136, *a*_1_ = 0.00543, *a*_2_ =  − 0.01207, *a*_3_ =  − 0.00195, *a*_4_ = 0.000413093, and *a*_5_ = 0.00177. Therefore, with |*m*| ≤ 15 the fitting results based on equation (6) were used, and the uncertainty is within 10% (uncertainty code 5 in HITRAN). For |*m*|> 15, a constant value 0.1440 corresponding to the average value of transitions with |*m*|= 15 was applied to the whole data set. And the value of 0.3481 was used for any unassigned lines. Furthermore, the temperature‐dependent exponent was set to 0.9 based on the average of the four measured transitions from Sur et al. (2016).

### 2.7 H_2_S

**Figure 8:**
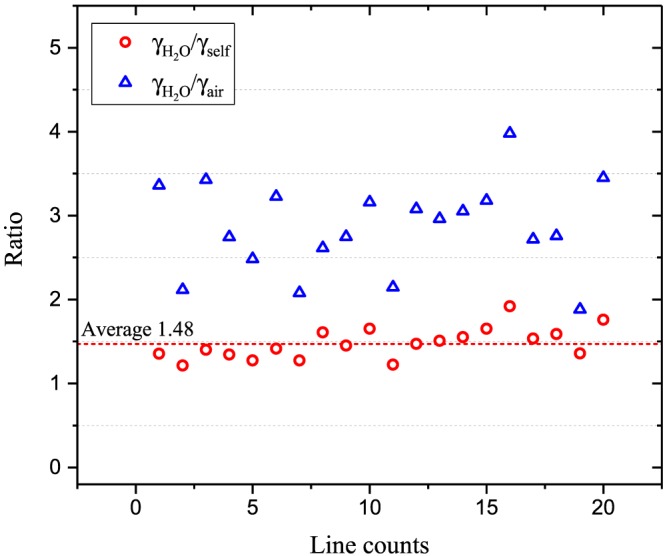
The ratio of γ_H2O_ from calculation (Starikov & Protasevich, 2006) to their corresponding self‐ and air‐broadening values of H_2_S lines from HITRAN (Gordon et al., 2017).

As mentioned in previous section, the experimental studies of ammonia opacities under Jovian conditions (Devaraj et al., 2014) determined the importance of accounting for broadening by water vapor. Combining this knowledge with the previous work (DeBoer & Steffes, 1994) it would be natural to assume that the same would apply for the studies of H_2_S. Additionally, since enhancement of H_2_S in human breath is linked to halitosis, it is important to know spectroscopy of hydrogen sulfide in humid conditions (Choi et al., 2014). While for H_2_S‐H_2_O there were no experimental data available in the literature, some calculations were available from Starikov and Protasevich (2006). The ratio of γ_H2O_ from calculation to that of self‐ and air‐broadening of H_2_S is shown in Figure 8. The derived scaling factor of 1.48 was used to scale the self‐broadening coefficients from the HITRAN database. The uncertainty is expected to be larger than 20% (error code 3).

## 3 Working With New Data Using HITRAN*online* and HAPI

Water‐vapor broadening HWHMs (γ_H2O_) as well as their temperature‐dependent exponents (n_H2O_) described above have been incorporated into the relational structure of the database and are already available via HITRAN*online* (Hill et al., 2016) and HAPI (Kochanov et al., 2016). Both these approaches in acquiring the HITRAN data were described in detail in corresponding papers and in the paper devoted to the HITRAN2016 edition (Gordon et al., 2017).

**Figure 9:**
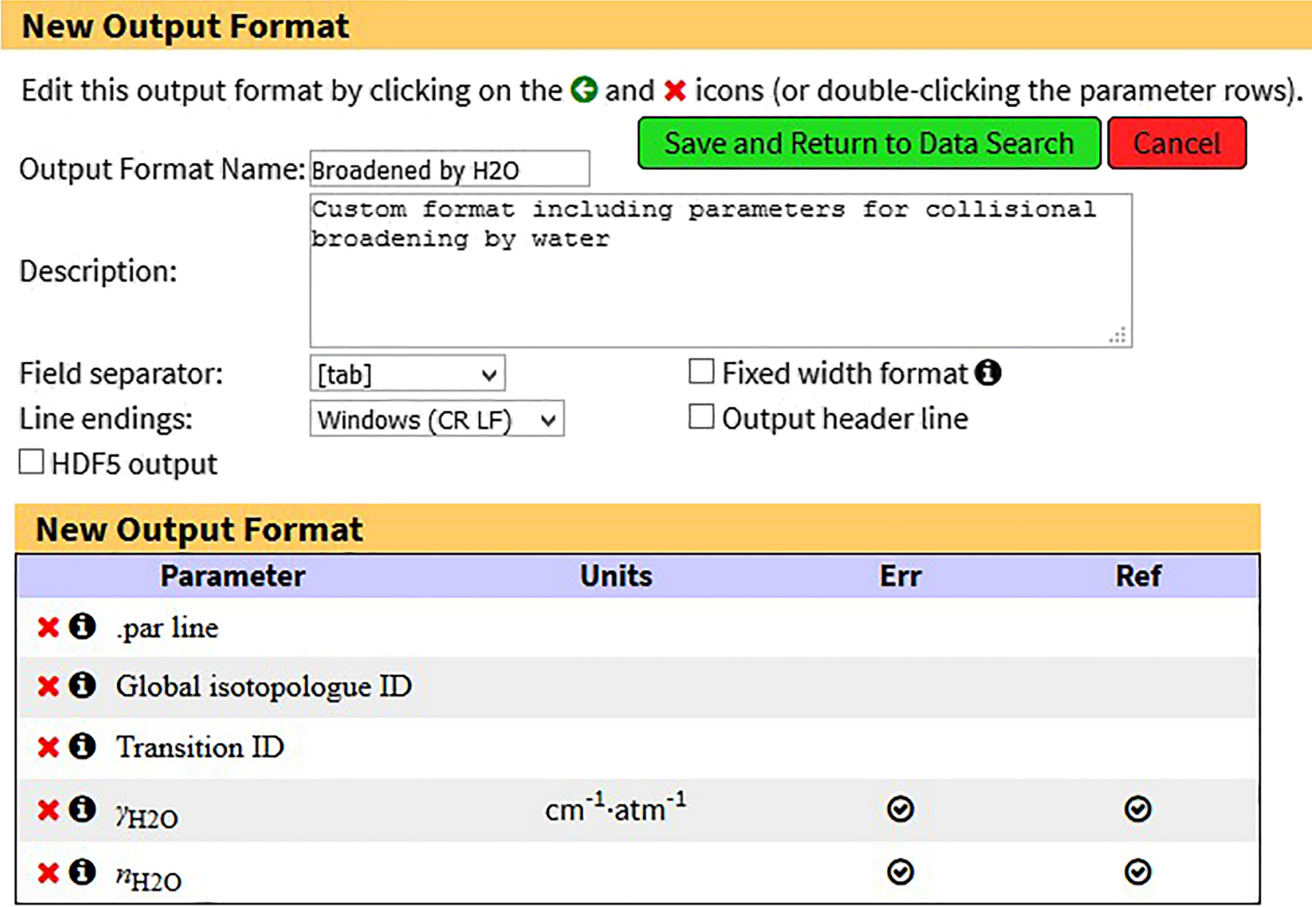
Sample custom output format creation at HITRAN*online* (https://hitran.org) for getting the water‐broadened lines. The line‐shape parameters (γ_H2O_ and *n*_H2O_) should be explicitly chosen from the parameter list to extract information relevant to this paper.

In HITRAN*online* (https://hitran.org), in order to get the extra line parameters discussed in this paper, the user needs to log in to the system and create a custom output format containing the parameters of interest. The details of this process are described in the dedicated paper by Hill et al. (2016). Figure 9 shows the example of such output format which includes γ_H2O_ and n_H2O_. By clicking on the corresponding checkboxes, it is also possible to request their error and reference codes.

In order to calculate absorption coefficients, absorption cross sections, transmission, etc. from the transitions using these foreign‐broadening parameters, the HITRAN Application Programming Interface (HAPI) tool is available. It can be obtained at the official web page (https://hitran.org/hapi) and on Github (https://github.com/hitranonline/hapi). HAPI is a set of Python libraries providing means to work with spectroscopic line lists, including calculation of different spectral functions. More details on its usage are given in the documentation which is available at the official HITRAN web page.

**Figure 10:**
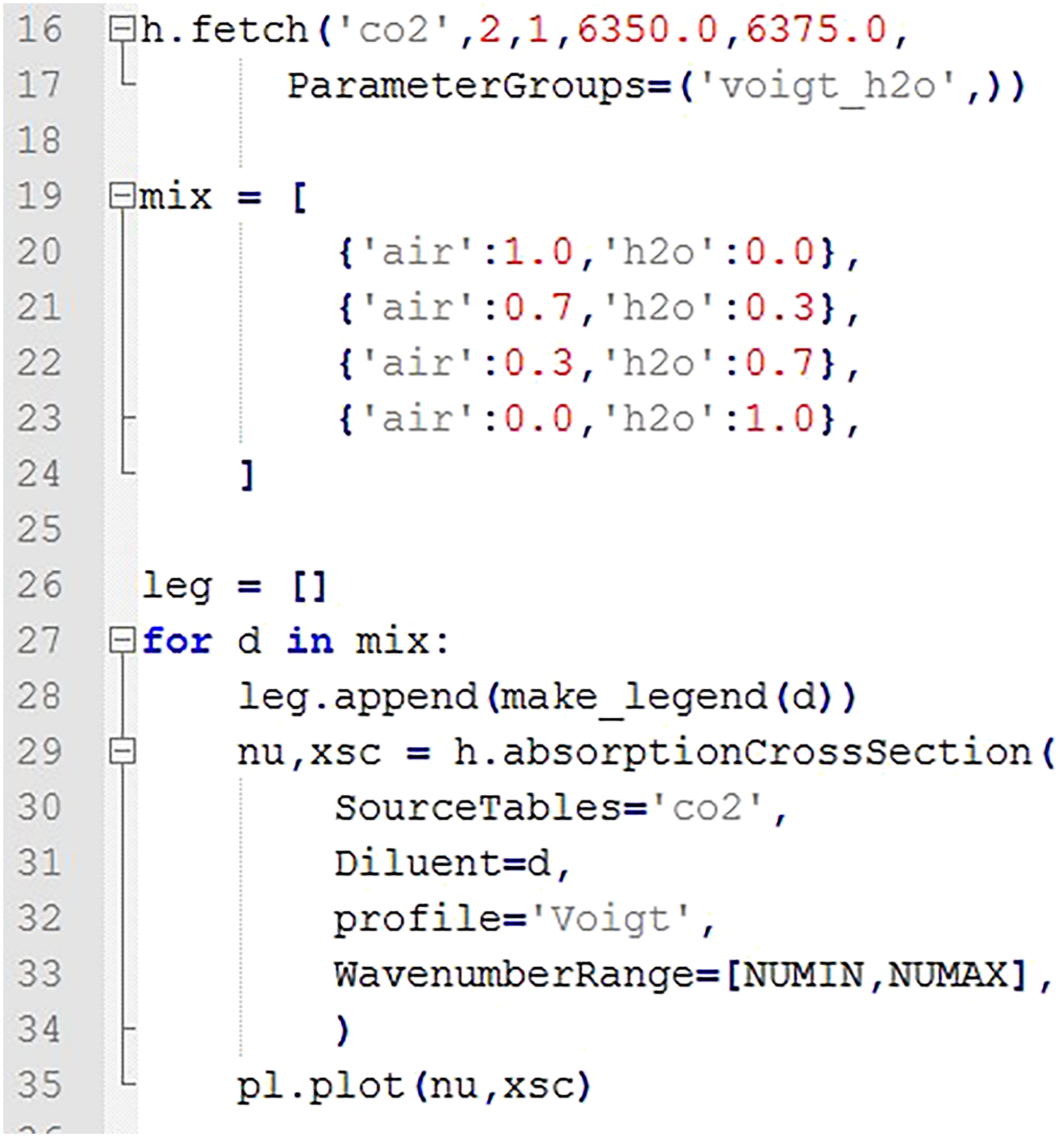
HAPI code snippet for calculating the absorption cross sections for CO_2_ lines in the 1.6‐μm spectral range using the Voigt line profile and four mixtures of air and water. The full code is available in the supporting information.

**Figure 11:**
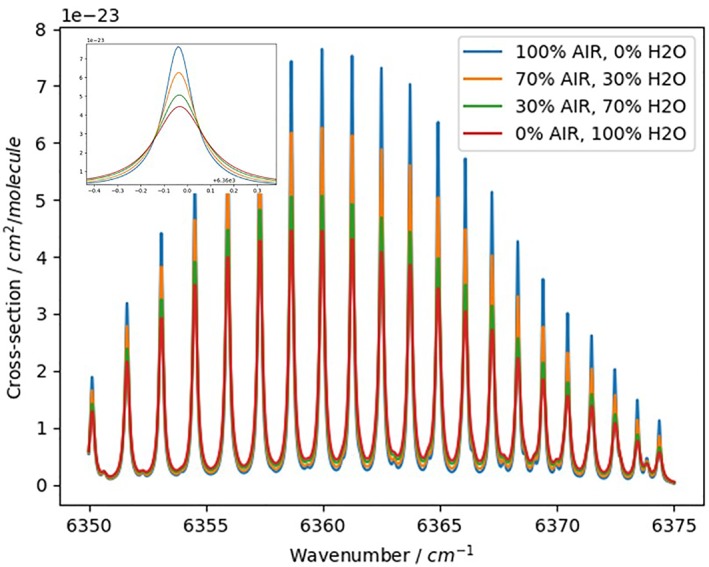
Result of the execution of the code given in Figure 10. The strongest line in this region is centered at 6,360 cm^−^ and zoomed in to show appreciable changes in the line shapes for different concentration of the broadeners (see legend).

Here we give an example which downloads the CO_2_ line list in the 1.6‐μm region accounting for the water broadening using the *fetch* function with explicitly specified “voigt_h2o” parameter group. The *absorptionCrossSection* function was used to calculate absorption cross sections from the downloaded line list. The code fragment corresponding directly to the calculation of absorption cross section is shown in Figure 10. The full code for downloading and calculating these sample spectra can be found in the supporting information. In the example given in Figure 11, we calculate and plot absorption cross sections for CO_2_ diluted in four different mixtures of air and water. Each mixture is passed to the function through the “Diluent” parameter, which is a dictionary of a type {‘air’:VMR_air_,‘h2o’:VMR_h2o_}.

It is worth noting that this approach works for the rest of the foreign broadeners since HAPI and HITRAN*online* follow the similar naming convention for the parameters (see Table 3 of Kochanov et al. (2016) and Table 3 of the HITRAN2016 paper (Gordon et al., 2017) for broadenings by CO_2_, He, and H_2_).

## 4 Conclusions

In this work the HITRAN database has been extended to include the water‐vapor broadening half‐widths as well as their temperature‐dependent exponents for CO_2_, O_2_, CH_4_, CO, NH_3_, N_2_O, and H_2_S based on semiempirical models. As shown in Table 3, the different numbers represent the data availability for each molecule in the present work. The ratio of the water vapor broadening to the air‐broadening was presented in Table 3 which also confirmed that water vapor is a more efficient broadener compared to air. It is clear that further studies are needed for some of these collisional systems.

**Table 3:** Data Availability for Broadening Coefficients (and Their Half‐Widths) of Spectral Lines of Different Molecules by Water Vapor

Parameter Molecule	γ_Η2Ο_	Ratio of γ_Η2Ο_/γ_air_	*n*
CO_2_	3	2.05	3
O_2_	3	1.11	2–3
CH_4_	1–2	1.36	1
CO	2–3	1.76	2–3
NH_3_	1–2	3.41	0
N_2_O	1	2.05	0
H_2_S	1	2.86	0

*Note*. 0 = no data available; 1 = few data available, new HITRAN file contains mostly averages; 2 = some measurements available, allowing semiclassical extrapolations; and 3 = relatively complete set of measurements or calculations available—at least for room temperature.

Every line for molecules from Table 3 in HITRAN now has the parameters in question. The data can be obtained through the HITRAN*online* interface and through HAPI as described in section 4. Procedures described here will allow estimating γ_H2O_ and n_H2O_ for lines beyond those that are currently provided in the HITRAN database. It is worth noting that HITRAN editions have supplied self‐broadening of water‐vapor lines. Thus, water‐vapor broadening of H_2_O lines was already provided, although without the corresponding temperature‐dependent exponents.

It is important to state that the work of adding line‐shape parameters associated with water vapor pressure to HITRAN is by no means complete. In the near future we plan to add these parameters for other HITRAN molecules (not targeted here). Also, once new measurements and calculations become available for molecules in Table 3, we will consider extending the work presented here.

At the moment there was not enough information to add shifts of spectral lines due to the pressure of water vapor. When a sufficient amount of measurements or calculations of the shifts becomes available, they will be added to the database. With that being said, these shifts will not make substantial differences in the spectral retrievals of the terrestrial atmosphere as shifts are generally from 1 to 2 orders of magnitude smaller than the widths (see for instance values in Delahaye et al. (2016c)) and at 4% concentration will constitute a very small contribution.

Acknowledgments

This work is supported by the National Aeronautics and Space Administration AURA (a NASA mission to study Earth's ozone) program grant (NNX17AI78G) and Planetary Data Archiving, Restoration, and Tools (PDART) program grant (NNX16AG51G). We also thank anonymous reviewers for constructive comments. The data generated in this paper are available on the HITRAN*online* website: https://hitran.org. Section 3 details how to retrieve and work with that data. Additionally, associated programs in Python that allow calculating the water‐vapor broadening parameters for CO_2_, CO, and O_2_ even at very high *J*'s are provided in the supporting information. In general, using procedures described here, researchers can estimate broadening coefficients (and their temperature dependencies) for any spectral line of gases in question (and their isotopologues) even if these lines are not in HITRAN.

## Supporting information

Supporting Information S1

Data Set S1

Data Set S2

Data Set S3

Data Set S4

Data Set S5

Data Set S6

Data Set S7

Data Set S8

Data Set S9
