# Supplementary material for: Introduction of Water‐Vapor Broadening Parameters and Their Temperature‐Dependent Exponents Into the HITRAN Database: Part I—CO2, N2O, CO, CH4, O2, NH3, and H2S
Source: J Geophys Res Atmos. 2019 Nov 7;124(21):11580–94. doi: 10.1029/2019JD030929 (PMC6919420; doi:10.1029/2019JD030929)
Supplement: Supplementary file 1 — Supporting Information S1 [file JGRD-124-11580-s001.docx]

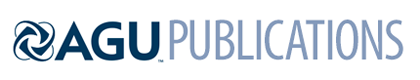


*[Journal of Geophysical Research: Atmospheres]*

Supporting Information for

**Introduction of water-vapor broadening coefficients and their temperature-dependence exponents into the HITRAN database, Part I: CO_2_, N_2_O, CO, CH_4_, O_2_, NH_3_, and H_2_S**

Y. Tan^1,2^*, R.V. Kochanov^1,3^, L.S. Rothman^1^, I.E. Gordon^1^*

^1^ Harvard-Smithsonian Center for Astrophysics, Atomic and Molecular Physics Division, Cambridge MA, USA.

^2^ Hefei National Laboratory for Physical Sciences at Microscale, iChem center, University of Science and Technology of China, Hefei, China.

^3^ Tomsk State University, Laboratory of Quantum Mechanics of Molecules and Radiative Processes, Tomsk, RUSSIA.

*Corresponding authors: Yan Tan ([tanyan@ustc.edu.cn)](mailto:tanyan@ustc.edu.cn)) ; I.E. Gordon ([igordon@cfa.harvard.edu)](mailto:igordon@cfa.harvard.edu))

**Contents of this file**

Summary of the Python codes and sample input and output files for water vapor broadening coefficients and their temperature dependencies for spectral lines of CO2, CO and O2.

**Introduction**

Supplementary material contains Python codes for calculating gamma_H2O and n_H2O of CO2, CO and O2 transitions in the HITRAN database as described in the article.

Sample input and output files are included. For CO2 it is just a fragment of the HITRAN2016 CO2 file, while for CO and O2 the entire HITRAN datasets are provided as an input. The program calculates new parameters and adds them (and their uncertainty codes) as extra columns to the traditional 160 character HITRAN format, in the following order

160 character line gamma_H2O Unc_ gamma_H2O n_H2O Unc_ n_H2O

Where uncertainty mappings are following HITRAN mapping given at: <https://hitran.org/docs/uncertainties/>

It corresponds to:

| 0 | Unreported or unavailable | | |
| --- | --- | --- | --- |
| 1 | | Default or constant |  |
| 2 | | Average or estimate |  |
| 3 | | ≥ 20 % |  |
| 4 | | ≥ 10 % and < 20 % |  |
| 5 | | ≥ 5 % and < 10 % |  |
| 6 | | ≥ 2 % and < 5 % |  |
| 7 | | ≥ 1 % and < 2 % |  |
| 8 | | < 1 % |  |

Note that the programs can be easily modified for any other input and output file formats.

**Dataset S1**.

**2019JD030929_CO2.py**

This is a Python code for calculating gamma_H2O and n_H2O of CO2 transitions in the HITRAN database.

**2019JD030929_CO2test.par**

This is an input file for the program above and essentially is an extract from the HITRAN2016 CO2 line list

**2019JD030929_CO2test.dat**

This is an output file for the **2019JD030929_CO2.py** program where gamma_H2O and n_H2O and their uncertainties are added as extra columns

**Dataset S2**.

**2019JD030929_CO.py**

This is a Python code for calculating gamma_H2O and n_H2O of CO transitions in the HITRAN database.

**2019JD030929_CO.par**

This is an input file for the program above and essentially is an entire HITRAN2016 CO line list for stable isotopologues

**2019JD030929_output_CO.dat**

This is an output file for the **2019JD030929_CO.py** program where gamma_H2O and n_H2O and their uncertainties are added as extra columns

**Dataset S3**.

**2019JD030929_O2.py**

This is a Python code for calculating gamma_H2O and n_H2O of O2 transitions in the HITRAN database.

**2019JD030929_O2.par**

This is an input file for the program above and essentially is an entire HITRAN2016 O2 line list for stable isotopologues

**2019JD030929_output_O2.dat**

This is an output file for the **2019JD030929_O2.py** program where gamma_H2O and n_H2O and their uncertainties are added as extra columns
